# Transforming community nursing services in the UK; lessons from a participatory evaluation of the implementation of a new community nursing model in East London based on the principles of the Dutch Buurtzorg model

**DOI:** 10.1186/s12913-019-4804-8

**Published:** 2019-12-09

**Authors:** Mirza Lalani, Jane Fernandes, Richard Fradgley, Caroline Ogunsola, Martin Marshall

**Affiliations:** 1 Department of Primary Care and Population Health, University College London, London, UK; 2 East London Foundation Trust, London, UK

**Keywords:** Community nursing, New models of care

## Abstract

**Background:**

Buurtzorg, a model of community nursing conceived in the Netherlands, is widely cited as a promising and evidence-based approach to improving the delivery of integrated nursing and social care in community settings. The model is characterised by high levels of patient and staff satisfaction, professional autonomy exercised through self-managing nursing teams, client empowerment and holistic, patient centred care. This study aimed to examine the extent to which some of the principles of the Buurtzorg model could be adapted for community nursing in the United Kingdom.

**Methods:**

A community nursing model based on the Buurtzorg approach was piloted from June 2017–August 2018 with a team of nurses co-located in a single general practice in the Borough of Tower Hamlets, East London, UK. The initiative was evaluated using a participatory methodology known as the Researcher-in-Residence model. Qualitative data were collected using participant observation of meetings and semi-structured interviews with nurse team members, senior managers, patients/carers and other local stakeholders such as General Practitioners (GP) and social workers. A thematic framework analysis of the data was carried out.

**Results:**

Implementation of a community nursing model based on the Buurtzorg approach in East London had mixed success when assessed against its key principles. Patient experience of the service was positive because of the better access, improved continuity of care and longer appointment times in comparison with traditional community nursing provision. The model also provided important learning for developing service integration in community care, in particular, how to form effective collaborations across the care system with other health and social care professionals. However, some of the core features of the Buurtzorg model were difficult to put into practice in the National Health Service (NHS) because of significant cultural and regulatory differences between The Netherlands and the UK, especially the nurses’ ability to exercise professional autonomy.

**Conclusions:**

Whilst many of the principles of the Buurtzorg model are applicable and transferable to the UK, in particular promoting independence among patients, improving patient experience and empowering frontline staff, the successful embedding of these aims as normalised ways of working will require a significant cultural shift at all levels of the NHS.

## Background

Health and care systems globally are facing the unprecedented pressures of increasing needs from an ageing population, rising workload for an overburdened workforce and limited financial resources [1, 2]. There is a growing consensus that integrated care is a key part of the approach to tackling these challenges with evidence that it may improve quality and outcomes, but more mixed evidence for value [3]. Establishing new models of care that enhance care coordination and promote a holistic, person-centred approach may increase patient satisfaction [4]. Some of these models of care promote service integration in community care with the primary aim of reducing hospital admissions by providing care closer to home [5]. Integration in community care is often characterised by multi-professional teams from across health and social care as well integrated services within community healthcare [6, 7]. In principle, integration promotes leadership among frontline care professionals [8]. Hence, models of community nursing care which include nurse led integration or coordination of care with other services, foster autonomous practice, empowering nurses to make independent decisions about patient care [9]. Such models are viewed as a key factor in improving the staff (and patient) experience [10].

### Community nursing and the Buurtzorg model

In several countries, the community care sector and in particular, nurses, play a key role in managing care for older people with chronic conditions at home, thereby reducing pressures on secondary care services [11]. However, retaining and recruiting nurses in community care is a pervasive problem due to a rising demand for nursing care at home and an ageing workforce [12]. Furthermore, increasing levels of work related stress and overly bureaucratised systems result in high levels of nursing staff turnover [13]. The Buurtzorg model has been reported as a possible solution to the challenges faced by community nursing services. The model is thought to improve both staff and patient experience while fostering professional autonomy through self-managing nursing teams [14].

Buurtzorg (Neighbourhood Care) was conceived in the Netherlands in 2006 at a time when the homecare industry was costly, fragmented and serviced by overworked community nurses, low on morale [15]. Buurtzorg is underpinned by an ethos of autonomous practice. Nurses lead the assessment, planning and coordination of patient care with autonomy in clinical decision-making, organising of work schedules and individual nurses determining their own professional development needs [16]. Other key features of the model include; relationship based practice, high levels of patient and staff satisfaction, financial sustainability, client empowerment and holistic care with a primary focus on clients with complex health and social care needs [17].

As a means of addressing some of the issues associated with the community nursing workforce, several countries have been piloting the Buurtzorg model, including the UK [18]. Buurtzorg has been successful in the Netherlands [15] which has a different health and social care landscape to that of the UK. Key differences between the two countries include the policy environment, funding mechanism and payment structure, which has prompted the Royal College of Nursing (UK) to caution against the simple transfer of the model [16]. Commentators have also suggested that the degree of autonomy afforded to nurses in the Dutch model would require a significant cultural and regulatory shift to current community nursing practice in the UK [16]. A first UK pilot of the model was undertaken by a National Health Service (NHS) Trust in South London. Findings from its evaluation indicated an improved patient experience when comparing a variant of the Buurtzorg model to routine community nursing services. Staff highlighted the autonomy afforded in clinical decision-making as a positive aspect of the model but also cited challenges, such as the limited recognition and support for self-management within a bureaucratic healthcare organisation [19].

### Local context

The London Borough of Tower Hamlets has similar problems to other parts of the UK with regard to understaffing in community nursing with a local vacancy rate of 21% [20]. Coupled with high levels of deprivation and some of the lowest levels of healthy life expectancy nationally, the Buurtzorg model provided Tower Hamlets with an opportunity to meet the needs of local service users with complex health and social problems [21, 22].

From June 2017 – August 2018, East London Foundation Trust (ELFT), an NHS community services and mental health provider, piloted a model of community nursing that adapted some of the principles of the Buurtzorg model (known locally as the Neighbourhood Care Team (NCT)). Adapted principles included a self-directed team (autonomy in clinical and operational decision making) with a flat hierarchy (team comprised of nurses with different levels of qualification), application of holistic care practices (provision of nursing and personal care) and low user caseloads (six patients to one nurse). Furthermore, the NCT aspired to deliver relationship based practice and promotion of user independence (empowering clients to manage aspects of their own care). Prior to the commencement of the pilot programme, the senior management team in ELFT in consultation with managers of the local community nursing teams and other stakeholders such as General Practitioners, determined these principles as being the most practicably implementable at the local level.

### The Neighboured care team

The NCT was located in a General Practice surgery in the southwest of Tower Hamlets serving a population of around 12,000, with the team’s caseload entirely comprised of patients from this surgery. The team included seven part and full-time community and district nurses with varying qualifications and experience (NHS band 5–7 nurses) and a band 4 healthcare assistant. Band 5 nurses are recently qualified whereas band 7 nurses have several years of nursing experience and may have previously held team lead roles [11]. To align with the Buurtzorg ways of working, there was no hierarchical structure in the team. The team was supported by a coach, whose role was to facilitate team dynamics, encourage dialogue and support cultural change. A senior district nurse (band 8) employed by ELFT was assigned the role of enabler (a new role not previously employed by Buurtzorg teams) to act as an operational interface between the team and the healthcare organisation. The NCT team was largely responsible for clinical and operational decision-making including managing team finance, administration and equipment procurement. The oversight and governance for the NCT pilot was managed by a steering group, which met monthly and comprised senior managers, the enabler and coach, patient involvement partner, the lead researcher (ML) and a Band 7 nurse from the NCT.

### The role of a Neighbourhood care team nurse

The NCT nurses were tasked to provide individualised patient care through holistic assessments. In addition to the provision of nursing care, the nurses would also deliver a Reablement service, which is not a formal component of community nursing provision in the UK. Reablement services, often provided by occupational therapists, offer short-term care at home, to assist recovery after discharge from hospital and involves supporting patients to regain the ability to undertake day-to-day activities such as dressing and preparing meals. It aims to increase the independence of a patient and is achieved through goal setting for the individual. In Tower Hamlets, Reablement care is normally provided by the local authority but for the patients on the NCT caseload, where appropriate, this service was provided by the nurses for an initial period of 6 weeks. The team planned to meet weekly with a social worker to review patients requiring Reablement support so as to determine the profile of the care package required.

It was expected that the patient caseload for the NCT would range between 40 and 60 patients at any one point during the pilot with nurses visiting between 4 and 6 patients a day. The number of patients seen daily, frequency of attendance and length of the visit would be determined by the needs of the patient, with some visits lasting over an hour where appropriate.

This study uses a participatory approach to evaluate the NCT model with the lead researcher embedded in the programme. It was thought that a participatory approach would facilitate the mobilisation of knowledge from both the published academic evidence as well as through the sharing of newly generated knowledge within the programme. The transfer of information between the NCT and the steering group could support the programme in achieving its objectives. This study aimed to examine the extent to which some of the principles of the Buurtzorg model could be adapted for community nursing in the UK.

## Methods

The evaluation was undertaken between July 2017 and September 2018. Interviews were conducted between June and September 2018.

### Study design

The Researcher in Residence model is an emerging approach to participatory research, which embraces the concept of ‘co-creating’ knowledge between researchers and practitioners [23]. In this study, the researcher (ML) was embedded in the pilot programme acting as an interface between the emerging evidence and its application to the service, co-creating knowledge through participation. Research expertise was communicated to and negotiated with the NCT team and programme steering group. The researcher’s primary role was to mobilise knowledge between the NCT team and steering group so as to support the programme in achieving its objectives. The evaluation was undertaken in a series of iterative stages of participation, data collection, analysis, interpretation and dissemination of emerging findings. Qualitative methods were used to generate and analyse the data and the findings presented are from field notes of meeting observations and interviews with key stakeholders.

To develop our understanding of the patient perspective we recruited a service user partner (JF) who was responsible for designing interview tools and undertaking interviews with patients/carers in receipt of NCT care, observing NCT meetings, analysing the data from both staff and patient/carer interviews and co-interpreting the findings.

### Ethics, consent and permissions

Ethics and governance approvals were provided by the NHS Research Ethics Committee (REC ref. 17/SC/0687) and the Health Regulatory Authority for an evaluation of the Tower Hamlets Together Vanguard programme of which the NCT pilot was a component. All, interview participants were approached by email or telephone by one of the researchers (ML and JF) who outlined the purpose of the study. Written informed consent was obtained from each participant prior to interview. Participants agreeing to interview returned their signed consent forms at the time of the interview. Participants were assured of confidentiality and anonymity and that participation was voluntary, and that they were free to withdraw from the study at any time. No participants withdrew their consent.

### Data collection

Participant observation was undertaken by ML and JF and involved attendance at monthly NCT steering group meetings and weekly NCT meetings amounting to around 40 h of observation. ML recorded field notes at meetings documenting aspects such as ongoing challenges, positive service developments, issues requiring managerial support and reflections on interactions between team members. As the evaluation progressed, in line with the participatory methodology, ML became an active participant in meetings, providing input and facilitating discussion [24]. Pertinent points arising from these observations were communicated to the NCT and steering group to facilitate discussion at future meetings and to enable them to determine how to use the findings in relation to the progress of the programme. We undertook semi-structured interviews (*n* = 23) with the NCT team and some of the steering group members. We also purposively selected stakeholders for interview who had worked with the NCT to obtain a range of perspectives about the pilot programme. Patients and carers were also purposively selected for interview from a patient list provided by the NCT. Having interviewed the majority of the key programme stakeholders and a sample of patients in receipt of the NCT service, the research team decided that no further interviews were required.

Interviews were conducted by ML, a researcher with experience of conducting health services research using qualitative methods and JF, a service user partner with experience of undertaking interviews as part of local service evaluations. Interviews with staff were held at the participant’s workplace in a private meeting room. Interviews with patients/carers were undertaken in the participant’s home. Interviews lasted between 30 and 70 min. All NCT and programme steering group members were known to the researcher (ML) and were aware of the purpose and aims of the study.

Interview guides (see Additional files 1 and 2) were formulated using relevant themes from the literature on models of integrated care as well as the Buurtzorg model. Guides were also informed by participant observation data. An inductive approach was taken with emerging themes from initial interviews used as a basis for further iterations of the interview guide. Interview guides for staff focussed on the implementation of the NCT model and comparisons with traditional community nursing. Interview guides for patients were about their experiences of care and how this differed from their previous experiences with traditional community nursing services where appropriate.

### Data analysis

Interviews were audio-recorded and transcribed verbatim. Data was managed using NVivo version 11.0. ML and JF conducted qualitative analysis using a thematic framework approach to code the data and identify patterns and themes [25]. A sample of transcripts were coded independently by ML and JF and the resulting themes were discussed to create a thematic framework. The framework was developed iteratively to capture emerging themes and was also informed by field notes from participant observation. Components of the analysis plan including co-interpretation of the findings was undertaken by three of the authors (ML, JF and MM). We formulated an organising framework for the data, constructed from the relevant academic literature.

## Results

### Participant characteristics

Semi-structured interviews were carried out with the NCT members (*n* = 7), the enabler, coach and two senior managers in ELFT as well as with twelve other stakeholders. These included; two GPs based at the surgery in which the NCT was co-located, two community nurses (external to the NCT) and a physiotherapist from the locality integrated care team, two social workers, a Reablement team manager and four patients/carers in receipt of NCT care. Two male and two female patients/carers aged 59–80 were interviewed. There were no refusals to participate among staff, but several patients were not contactable (did not answer the phone or had moved from the recorded residential address). Two patients/carers did not want to participate but did not provide reasons for not doing so.

### Key themes

The findings presented here are primarily from field notes from participant observation of meetings and interviews with key stakeholders. Emerging themes from the data were organised using the framework under three headings; integration of the NCT with local health and social care services, self-management of the NCT and impact of the wider health and social care context.

#### Integration of the NCT with health and social care services

A key enabler for the model was the successful integration of the NCT with local health and social care services. The NCT appeared to form good working relationships with several local stakeholders. In particular, they formed an effective working partnership with staff from the GP surgery within which they were co-located, developing a relationship based on mutual respect and trust, both of which are thought to facilitate collaboration between care professionals [26]. The closer working reflected goodwill on part of both the NCT nurses and the practice staff [27]. Indeed, the NCT nurses remarked on the ‘open door’ policy advocated by GPs which facilitated face-face communication. The GPs interviewed also highlighted the positive impact of the NCT on local health service outcomes.

*‘An example of somebody who I went to visit…. It was Friday evening she didn’t speak English, completely housebound and I was unable to get hold of Social Services. Had we not had the NCT I almost certainly would have had to admit her (to hospital) just from a social thing. I spent two hours on the phone to the Social Services and... I couldn’t get hold of anybody. But because I knew that the NCT would see her that night or the next morning and sort everything out, I didn’t admit her. I spoke to them on Saturday morning and they sorted her out… they got the food in, they got social services in…’* General Practitioner

#### Self-management of the NCT

A key feature of the Buurtzorg model is autonomous practice which we explore below.

##### Contrasting features of the NCT model and traditional community nursing

Overall staff and patients suggested their experiences of delivering and receiving care had improved when comparing the NCT model to traditional community nursing. This was for three reasons. Firstly, most interviewees suggested that delivering a model of care that enabled nurses to spend extended periods of time with patients focussing on promoting self-care and independence, were aspects of the model that were viewed favourably by staff and patients. The NCT suggested that due to traditional community nursing being task orientated, the holistic Buurtzorg ways of care delivery fostered better quality and more patient-centred care.

*‘Someone described that they felt ‘held’ by us… a patient who is terminally ill and we spent a lot of time with them. They were seen by other district nurses and just felt ‘rushed’ and sort of ‘invaded’ …So I think they felt like we made that as gentle as possible.’* NCT nurse

Secondly, flexibility which enabled nurses to manage their own work schedules, was mentioned as a key feature of the NCT model and proposed as a significant driver for applying to be a NCT nurse. The nurses contrasted the flexibility in the NCT model with traditional community nursing which they viewed as less likely to provide an adequate work/life balance due to high vacancy rates and low staff retention, which resulted in staff being overworked.

Thirdly, the findings suggest a positive patient/carer experience of the NCT service. In addition to the extended appointment time, patients/carers remarked on the accessibility to the NCT team when compared to routine community nursing services. For example, patients could contact the NCT directly on the telephone between 8 am and 8 pm. Patients also highlighted the continuity of care as an important feature whereby each NCT nurse had a comprehensive understanding of the patient’s needs creating a sense of familiarity that was lacking in interactions with traditional community nursing services.

*I think because they (NCT) were the only ones that used to come, you get to know them … I knew at eight o’clock when I rang they would get back to me and they would sort mum out. You do feel that little bit of closeness…with the nurses we have got now, you don’t know who is coming.* Carer

##### Barriers to autonomous practice

Nurses mentioned that they had autonomy in clinical decision-making but felt that they were seldom able to practice full autonomy in operational decision-making. They suggested this was because the organisation showed reluctance in relinquishing responsibility over some aspects of the service e.g. management of the programme budget. Yet, senior management believed they had adequately supported the NCT nurses to take on various clinical and operational leadership roles. Furthermore, the coach and enabler roles were partly established to support the team to work autonomously.

*‘Can the team exercise a sense of autonomy and feel confident in doing that? Having the permission from the organisation to do that, they did have the permission, but it’s just the sense that they’ve struggled with grasping that? And I think they had the perception that the organisation was struggling to let go of control?’* Senior manager

##### Team dynamics

Team dynamics were a barrier to the development of an effective working relationship among the NCT nurses. The nurses were recruited through non-standardised approaches including an assessment day conducted by individuals both associated with and external to, the programme. Despite this innovative approach to recruitment, the appointed nurses expressed different aspirations and expectations of working as a NCT nurse. Additionally, creating a nursing team with a flat hierarchy comprising nurses with varying qualifications and experience, appeared to hinder the development of effective working relationships between some of the NCT members.

*‘The flat hierarchy is going to take a long time for nurses to get used to. If I’m a Band 7 and I’m used to commanding you it is very difficult for a Band 4 to tell me what to do. So, it is that power struggle psychologically and I’ve heard that senior nurses are moaning about it.’* NCT team member

*‘The flat hierarchy is causing problems - there is need for open dialogue and for the junior nurses to assert their opinions to benefit the whole team, but ultimately its simpler for the nurses to revert to their preordained roles prior to joining the NCT.’* ML field notes

#### Impact of the wider health and social care context

Various aspects of the UK care system limit the direct adoption of the Buurtzorg model and these are presented below.

##### System pressures

A key issue raised during interviews was of system pressures and specifically the patient caseload. While the caseload at the beginning of the pilot was close to the level expected of a Buurtzorg team (6:1), at times it was much higher for the NCT rising to 10:1. When the caseload was high, the nurses felt overburdened which had a negative effect on team morale.

*‘And then suddenly they went from managing a small caseload quite well and had bit of a space to really think about what they were doing, to suddenly it being ramped up and then the pressure built up.’* NCT stakeholder

##### Health system barriers

Two key health system barriers affected the successful implementation of the model. Firstly, some interviewees believed that the national and local profile of the programme, increased scrutiny on the team and may have engendered a higher level of oversight, which was conflated by the nurses with additional administrative burden. A principle of the Buurtzorg model is the streamlining of administrative functions thereby reducing bureaucracy. The NCT were provided with a substantive ‘back office’ function, partly administered by the team enabler who assisted the team in accessing support for human resources, IT and procurement, from within the organisation. Interviewees from the management team suggested that the nurses possibly created extra work for themselves because of not accessing the relevant support and training functions provided by the organisation. Hence, the additional work ascribed by the NCT as ‘administrative’ tasks such as managing team rotas, equipment procurement, etc. were activities that Buurtzorg nurses would be expected to undertake routinely.

Secondly, while most interviewees believed that clinical competency was fundamental to the role of any nurse, some suggested that NCT nurses should also have good IT skills as the Buurtzorg model promotes mobile working and IT proficiency. All nurses were assigned different roles within the team which were periodically rotated during the pilot to ensure that each nurse was exposed to the non-clinical activities of the NCT. Nonetheless, only two of the nurses felt confident in undertaking the non-clinical tasks resulting in them feeling overworked, due to managing most of the administrative tasks for the entire team.

*‘I think a lot of the admin has fallen on myself and [name] because we’re good with computers… And Buurtzorg place huge importance on making the most of technology and trying to be ‘paper light’…..But there’s a reluctance to let things go. People still want to use paper annual leave cards. We’ve got software that does this.’* NCT nurse

##### Role of the NCT model in future community care service planning

Some interviewees suggested that the purpose of the programme was less about redesigning community nursing based on the Buurtzorg model, and more about exploring the adoption and adaptation some of the model’s principles, such as promoting independence among patients and reducing duplication of services. An example of reducing duplication was the Reablement service provided by the NCT which reduced the workload of Reablement services, providing potential cost savings in another part of the care system.

*‘District nurses and carers turning up at the same time, I’ve seen this many times... doing similar tasks and it’s costing two authorities…cracking that properly was the big prize out of this. And that’s without even talking about the benefits for the patient. So that self-management stuff, the person-centred care.’* Senior manager

## Discussion

### Summary

This qualitative study has provided new insights into the extent to which some of the Buurtzorg principles can be adapted for community nursing in the UK. Broadly, implementing the model as it is employed in the Netherlands would be challenging in the UK due to prevalent cultural and systemic barriers in the NHS. The study found that the nursing staff experience was variable with increased satisfaction from managing their own work schedules and providing less task orientated care, offset by organisational pressures (the increasing of the patient caseload) and a perceived greater administrative burden. The findings also suggest that autonomous practice among frontline staff warrants further consideration as even though senior managers advocated greater autonomy and empowered the NCT, the nurses felt unable to enact it in practice. The study also found that the patient/carer experience was largely positive and that the NCT succeeded in developing effective collaborations across health and social care, particularly with GPs.

This study identified several lessons from the pilot programme that are relevant to the development of service integration in community care services and may affect patient and service outcomes. Firstly, two of the key principles of Buurtzorg, promoting independence among its patients and the provision of personalised care, align with the ethos of integrated care and feature strongly in the NCT model [26]. Secondly, reducing care duplication through better care coordination and the provision of a Reablement service reduced workload in another part of the care system. Thirdly, if NHS organisations are committed to improvement and exploring models of care in which service delivery focusses on relational care, they must create an environment in which new initiatives can develop without succumbing to system pressures**.** In this study, a larger than initially expected patient caseload overburdened the team, hampering the ability of the nurses to deliver the quality of care to which they aspired. Fourthly, recent centralisation of community health services in the UK [28] has resulted in less co-location with general practice and hence, reduced face-face dialogue between community care professionals and GPs. In this study, GPs provided several examples of the benefits of co-location especially effective communication with the NCT through frequent and informal face-to face discussion, which was thought to lead to improved patient outcomes. Finally, patient satisfaction has previously been described as a key indicator of the quality of care and is an underpinning principle of the Buurtzorg model [18, 29]. In this study a positive patient experience was as a result of the NCT model replicating aspects of the Buurtzorg approach [19]; improved accessibility, continuity of care and extended appointment times were mentioned as aspects that distinguished the model from traditional community nursing.

Aside from the evaluation of a community nursing model based on Buurtzorg in South London, there is limited available information from the several Buurtzorg models operating in other countries [18]. However, parallels can be drawn with the *Drennan* et al study, as both found an improvement in the patient experience (due to continuity of care) as well as perceptions among the nurses of limited autonomy in operational decision-making [19]. Our study builds on these findings, providing novel insights into the implementation of some aspects of the Buurtzorg model. For example, we found that while senior leaders championed autonomous practice, an embedded professional hierarchical culture made it challenging for nurses working in a small scale, time-limited pilot in a large healthcare organisation, to fully understand and adopt autonomous practice [30]. Moreover, the development of an effective working relationship among NCT nurses may have also been limited by the engrained hierarchical tradition within nursing, as it appeared difficult for senior nurses to shift an established mind-set in which they may view junior nurses as subordinates to perceiving them as equal partners.

Also, associated with effective self-management is the need for Buurtzorg nurses to have good IT and administrative skills especially as Buurtzorg is an exponent of mobile working. In this study, the findings identified a need for building skills capacity in IT and administration among the NCT nurses. The skills gap identified in this study may not be ubiquitous issue in the UK, but successful implementation of a community nursing model based on Buurtzorg may require adapting complex administrative processes to reduce bureaucracy and upskilling of nursing staff to capitalise on recent advances in technology and digitisation.

### Strengths and limitations

A strength of this study was the participatory approach to the evaluation which aided the development of the programme through mobilisation of published academic evidence, sharing of newly generated knowledge to support the programme to meet its objectives, and the transfer of information between the NCT and the steering group. However, some aspects of the evaluation were more participatory in nature (co-design of the study, discussion and co-interpretation of findings with the steering group) than others (ongoing discussion of findings with the NCT). There were limited opportunities to co-produce recommendations with the NCT or steering group due to the programme ending before fieldwork was completed.

An in-depth focus on a single pilot of a model based on the Buurtzorg approach in an individual GP surgery may not produce generalisable findings but as yet, this is only the second published study in a UK context and the lessons from this evaluation do provide some transferable learning. The study did not assess the impact of the NCT service on secondary and primary care outcomes nor did it explore the economic implications to the care system.

As a result of the small sample of patients/carers recruited to the study, the data from these interviews may not provide a representative view of all patients in receipt of care from the NCT. However, the data obtained from patients/carers provided a nuanced, detailed and novel perspective reaffirming the positive experience of patients in receipt of Buurtzorg modelled care in other studies [14, 19]. Furthermore, the patient/carer interview data also reflects the reported positive experiences of the NCT patients relayed through interviews and observations with other stakeholders (e.g. GPs) during the course of the study.

## Conclusion

Our findings suggest that it may prove challenging to directly transfer the Buurtzorg model to the UK health system. However, there are some principles which are adaptable, or could be aspired to, within community nursing. Promoting greater independence among patients while improving access and continuity of care, more flexible working for community nurses, forming effective inter-professional partnerships and empowering frontline staff are all adaptable principles of the Buurtzorg model that ought to be prioritised as part of community care development. Some of these principles also align with integrated care approaches and combining them with the provision of Reablement services and care-coordination present in the NCT model evaluated here, provides important learning for the development of service integration in community care.

At a time when workforce pressures and constrained resources are prevalent, applying some of the Buurtzorg principles will likely improve patient care in the community. The successful embedding of these principles as normalised ways of working will, however, require a cultural shift at all levels of the care system in the UK and hence, should form a key component of organisational development strategies for health and care organisations.

## Supplementary information

**Additional file 1.** Interview guide; NCT staff and other key stakeholders.

**Additional file 2.** Interview Guide; patients/carers in receipt of NCT service.

## Data Availability

The datasets generated and/or analysed during the current study are not publicly available. This is due to the participatory approach to the study with the data potentially containing information that could compromise the research participants.

## Abbreviations

ELFT: East London Foundation Trust
GP: General Practitioner
NCT: Neighbourhood Care Team
NHS: National Health Service
