# Supplementary material for: Transforming community nursing services in the UK; lessons from a participatory evaluation of the implementation of a new community nursing model in East London based on the principles of the Dutch Buurtzorg model
Source: BMC Health Serv Res. 2019 Dec 9;19:945. doi: 10.1186/s12913-019-4804-8 (PMC6902511; doi:10.1186/s12913-019-4804-8)
Supplement: Supplementary file 1 — Additional file 1. Interview guide; NCT staff and other key stakeholders. [file 12913_2019_4804_MOESM1_ESM.docx]

### **Supplementary File 1**

### **Interview guide; NCT staff and other key stakeholders**

Thank interviewee for agreeing to participate and making the time. Remind interviewee of purpose of interview. Check information sheet has been read, consent form signed and audio recording has been agreed. Any questions or clarifications before commencing interview?

Background

Tell me about your current role.

What is your role in the NCT programme?

How did you (or your organisation) become involved in the NCT programme?

Why did you apply for the position of an NCT nurse (where applicable)?

Programme content

What aspects of the NCT model have worked well?

- *Compare to traditional community nursing.*
- *System benefits?*
- *Patient/user satisfaction?*
- *Benefits to everyday practice?*

What aspects of the NCT model have you found challenging? Why?

Please provide suggestions as to how some of these issues could be addressed?

*Nurses only:* To what extent have these issues been addressed by senior management?

Describe a notable experience you had when delivering care as a NCT nurse?

- *What has been the overall impression of the service from patients/carers/family members?*

Adoption of Buurzorg principles

Which of the principles of Buurtzorg have you adopted (and adapted)?

For those principles not adopted, why have you not adopted these?

- *Barriers to adoption?*

Comparison with traditional community nursing

Tell me about how the NCT model compares to traditional community nursing services.

- *Which is preferred and why?*
  - Patient experience
  - Staff experience
  - Patient outcomes
  - Financial considerations

Specific questions (if not covered above)

Tell me about your experience of the self-direction/management aspect of the NCT?

Future of NCT model

What is the future of the NCT model as part of the local community care provision?

If you choose not to scale up the model, which principles of the NCT would you still consider adapting for community care locally?

Have you any questions for me?
