# Supplementary material for: Transforming community nursing services in the UK; lessons from a participatory evaluation of the implementation of a new community nursing model in East London based on the principles of the Dutch Buurtzorg model
Source: BMC Health Serv Res. 2019 Dec 9;19:945. doi: 10.1186/s12913-019-4804-8 (PMC6902511; doi:10.1186/s12913-019-4804-8)
Supplement: Supplementary file 2 — Additional file 2. Interview Guide; patients/carers in receipt of NCT service. [file 12913_2019_4804_MOESM2_ESM.docx]

### **Supplementary file 2**

### **Interview Guide – patients/carers in receipt of NCT service**

Thank interviewee for agreeing to participate and making the time. Remind interviewee of purpose of interview. Check information sheet has been read, consent form signed and audio recording has been agreed. Any questions or clarifications before commencing interview?

Care experience

How long have you/the person you care for been receiving care in the home?

If, you are happy to, please describe the reasons for you/ the person you care for receiving care at home.

You have been receiving care from a new nursing service here in Tower Hamlets. Please describe your experience of the care provided by the nurses.

Tell me how the nurses support you.

To what extent have you/the person you care for views been taken into account when making decisions about your care?

Aspects of care

Aside from providing basic nursing care e.g. changing dressings etc. please describe some of the other tasks the nurses have carried out.

Have you received care from nurses in Tower Hamlets in your home before?

- If yes, how does the service provided by this team of nurses differ (if at all)?

What aspects of the service have worked well for you/the person you care for?

Is there anything you would change about the service?

- *If so, what would that be?*

What impact has the service had on you/the person you care for daily life?

Tell me about how confident you feel in managing your own care following your time as patient in receipt of the NCT service?

Have you any questions for me?
